# Molecular cytogenetic characterization of undifferentiated embryonal sarcoma of the liver: a case report and literature review

**DOI:** 10.1186/1755-8166-5-26

**Published:** 2012-05-03

**Authors:** Xiaoxia Hu, Haiying Chen, Meishan Jin, Xianfu Wang, Jiyun Lee, Weihong Xu, Rui Zhang, Shibo Li, Junqi Niu

**Affiliations:** 1 Department of Internal Medicine, The First Hospital of Jilin University, Jilin 130021, China; 2 Department of Pediatrics, The University of Oklahoma Health Sciences Center, Oklahoma, OK 73104, USA; 3 Department of Pathology, The First Hospital of Jilin University, Jilin 130021, China; 4 Department of Pathology, College of Medicine, Korea University, Seoul, South Korea; 5 Department of Hematology, The First Hospital of China Medical University, Liaoning 110001, China

**Keywords:** Undifferentiated embryonal sarcoma of the liver (UESL), Cytogenetic anomalies, FISH, aCGH, *TP53* mutation

## Abstract

Undifferentiated embryonal sarcoma of the liver (UESL) represents a heterogeneous group of tumors derived from mesenchymal tissues. Earlier cytogenetic studies in limited cases demonstrated that UESL is associated with a recurrent translocation t(11;19)(q11;q13.3-q13.4) or add(19)(q13.4). In this report, we present our array comparative genomic hybridization (aCGH), fluorescence *in situ* hybridization (FISH) findings, and a missense mutation of *TP53* gene by DNA sequencing in a 19-year-old patient with UESL. The data were compared to laboratory findings reported by previous studies.

## Background

Undifferentiated embryonal sarcoma of the liver (UESL) is very rare and highly malignant. It occurs predominantly in children with a peak incidence in the age range of 6–10 years without sexual predomination [1]. Modern supportive therapy and multimodal treatment, including tumor resection and adjuvant chemotherapy, have improved survival [2]. UESL is sometimes misdiagnosed as other types of sarcomas involving the liver [3], including as a poorly differentiated or sarcomatoid variant of hepatocellular carcinoma [4] or as benign hepatic lesions [5]. Currently, oncogenesis of UESL remains uncertain. Multiple cytogenetic studies of sporadic cases demonstrated that UESL frequently harbors chromosomal rearrangements of 19q13.4, including the translocation t(11;19)(q11;q13.3/13.4) [6] and add(19)(q13.4) [7]. Sowery et al. used a conventional CGH technique to investigate chromosomal imbalances in six patients with UESL and reported losses or gains of whole chromosomes or partial chromosomal regions [8]. Using DNA sequencing analysis, the breakpoint of the translocation t(11;19) on 11q was detected in the *MALAT1* gene, i.e., the *MALAT1* gene was rearranged at t(11;19) [9]. Point mutations of the *TP53* gene were also detected in three UESL cases [10,11]. To our knowledge, we are the first to use high-resolution array-CGH analysis (aCGH) to detect more subtle segmental genomic imbalances, which were followed by fluorescence *in situ* hybridization (FISH) to confirm some of the selected anomalies identified by aCGH. In addition, given that a hemizygous deletion of the *TP53* gene was identified in our case by aCGH and FISH, subsequent DNA sequencing was performed to identify additional gene mutations in the rest of the *TP53* allele in this case.

## Case presentation

A 19-year-old girl was admitted to the Surgical Department of the First Hospital of Jilin University because of continuous abdominal distension and increasing abdominal girth. An abdominal ultrasound revealed the presence of a lesion in the right lobe of the liver (approx. 19 × 17 cm). Computed tomography revealed a well-defined, low-density mass in the right lobe. The initial impression by the CT scan was possible hepatic echinococcosis. Laboratory investigations showed a normal serum α-fetoprotein level, normal albumin, normal aspartate aminotransferase (AST), and normal alanine aminotransferase (ALT). Due to the good general condition of the patient and the history of close contact with pets, the primary diagnosis was hepatic echinococcosis. An open biopsy of the liver mass was performed, and subsequently, the patient underwent right hemihepatectomy.

**Figure 1:**
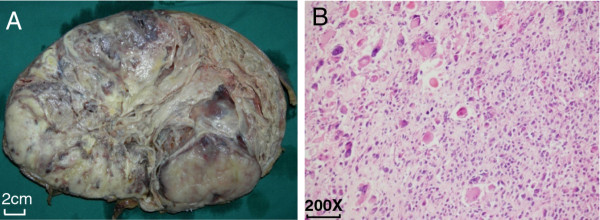
**Tumor morphology. (A)** Resected specimen. **(B)** Pleomorphic lesion with an abundance of bizarre cells, including multinucleated cells and others with eosinophilic inclusions in the cytoplasm on the left upper corner; primitive undifferentiated fusiform cells on the right lower corner.

The gross specimen weighed 3200 g and measured 25 × 19 × 12 cm. The lesion was well demarcated from the surrounding hepatic parenchyma by a fibrous pseudocapsule. The tumor had multiple greyish-white, partially mucoid, fluid-filled cysts together with areas of hemorrhage and necrosis (Figure 1A). On microscopic examination, there was no hydatid or hydatid scolex in the fluid. The tumor was composed of a pleomorphic lesion with an abundance of abnormal cells, including multinucleated cells, primitive undifferentiated mesenchymal cells, and fusiform cells (Figure 1B). Immunohistochemically, the tumor expressed vimentin and macrophage-myeloid associated antigen (CD68) but did not express actin, epithelial membrane antigen (EMA), smooth-muscle actin (SMA), or α-fetoprotein. The pathological diagnosis was undifferentiated embryonal sarcoma of the liver with resection margins free of disease. Magnetic resonance imaging (MRI) demonstrated no evidence of disease one month post-surgery. Forty months post-surgery, the girl remained clinically well.

## Materials and methods

### Oligonucleotide aCGH assay

Genomic DNA was extracted from the patient’s paraffin-embedded tumor tissue block using the QIAamp DNA FFPE tissue kit (QIAGEN, Hilden, Germany) according to the manufacturer’s protocols. Human genomic reference DNA was purchased from Promega (Promega Corporation, Madison, WI, USA). The sonicated patient DNA and reference DNA were labeled with either cyanine 3 (Cy-3) or cyanine 5 (Cy-5) by random priming (Trilink Biotechnologies, San Diego, CA, USA). These samples were subsequently hybridized to a NimbleGen high-capacity 385 K oligo microarray chip (Roche/NimbleGen System Inc., Madison, WI, USA) by incubating in a MAUI Hybridization System (BioMicro Systems, Salt Lake City, UT, USA) for 18 h according to NimbleGen’s CGH protocols. The array was scanned at 532 and 635 nm using the GenePix scanner (Molecular Devices, Sunnyvale, CA, USA). NimbleScan and SignalMap (NimbleGen System Inc, Madison, WI, USA) were applied for data analysis.

### Fluorescence *in situ* hybridization (FISH)

FISH analysis was performed on paraffin slides cut from the paraffin-embedded tumor tissue using multiple DNA probes, including the locus-specific probes LSI D5S21/EGR1, LSI c-MYC, LSI IGH/FGFR3 and LSI TP53. All the probes were purchased from a commercial source (Abbott Molecular Inc., Des Plaines, IL, USA) and were used according to the manufacturer’s protocols with minor modifications.

### DNA sequencing analysis of the *TP53* gene mutation

Polymerase chain reaction (PCR) was performed using primers designed to target exons 2–11 of the genomic DNA of the *TP53* gene (GenBank accession number *NM_000546.4*). Mutation nomenclature follows the numbering recommended by the Human Genome Variation Society with +1 nucleotide as the A of the ATG initiation codon [12]. The 25 μl PCR mixture contained 50 ng of template DNA, 1X PCR buffer, 0.2 mmol/L dNTP mix, 50 ng of each primer, 2.5 mmol/L MgCl_2_ and 1 unit of AmpliTaq Gold (Applied Biosystems, Foster City, CA, USA). The ddNTP terminator reaction was carried out with ABI BigDye Terminator v3.1 Cycle Sequencing kit (Applied Biosystems). The data were collected on an ABI 3130xl Genetic Analyzer (Applied Biosystems). Mutation Surveyor (SoftGenetics, State College, PA, USA) was the primary tool used in the data analysis.

## Results

**Figure 2:**
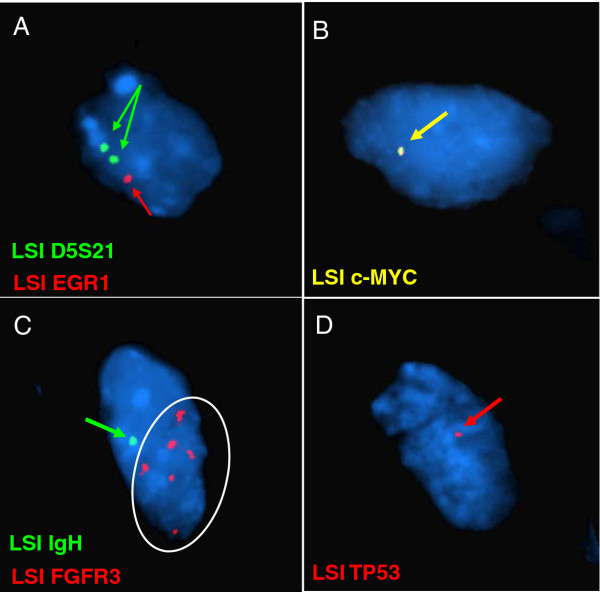
**FISH analysis using selective probes confirmed the aCGH results. (A)** It showed only one signal of the LSI EGR1 (red) and two signals of LSI D5S21 (green), indicating a loss of the *EGR1* gene. **(B)** One signal of the LSI c-MYC (yellow) demonstrates a loss of the *MYC* gene. **(C)** One signal of the LSI IGH (green) indicates a loss of the *IGH* gene, and the white circle indicates amplification of the *FGFR3* gene (red). **(D)** One signal of the LSI TP53 indicates a loss of the *TP53* gene.

**Figure 3:**
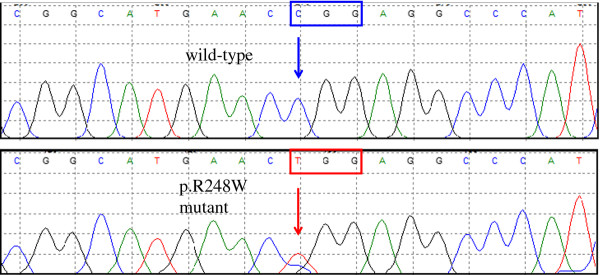
**A point mutation (C>T) in *TP53 *gene at nucleotide 13379 (g.13379 C>T) resulting in the substitution of an arginine for a methionine at codon 248 (R248W) was detected in the tumor tissue.**

The losses and gains of chromosomal material as well as their specific genomic sizes, as detected by aCGH, are presented in Table 1. The tumor had losses of multiple chromosomal regions, including 1p13.1, several regions on 2q, 3p, 4q, and alternating deletions on 5q, 8q24.12-qter, 9p24.1, 9q34.11, 9q34.3, 10pter-q22.1, 11pter-p15.4, 14q12-qter, 15q, 16pter-p13.3, 17pter-p12, 19p13.3-13.11, 20q13.32-qter, 22q12.3, and 22q13.1-qter. The tumor had gains on 1pter-p36.33, 2pter-p25.3, 2q14.1, as well as several regions on the short arm of chromosome 4, 4q31.1, 5pter-p15.31, 5q34, 5q35.2-qter, 6q22.33, 19p13.11, 20q11.1-q13.32, 21q22.13-q22.3, and 22q13.1. To confirm a selection of the tumor-related regions detected by the aCGH assay, FISH was performed using multiple corresponding DNA probes. A total of 200 cells were analyzed for each probe used. Approximately 80% of cells analyzed had either a loss or gain of regions including *EGR1* (5q31.2), *MYC* (8q24.21), *IGH* (14q32.2) and *TP53* (17p13.1) as well as a gain of *FGFR3* (4p16.3) (Figure 2A-D). A point mutation (C>T) in *TP53* gene exon 7 at nucleotide 13379 (g.13379 C>T) resulting in the substitution of an arginine for a methionine at codon 248 (R248W) was identified in this case (Figure 3).

**Table 1:** Summary of genomic imbalances detected by aCGH in this case

**Chromosome region**	**Genomic coordinates (NCBI Build 36.3) (bp)**	**Size (Mb)**		**Number of genes (Interesting genes)**
		**Loss**	**Gain**	
1pter-p36.33	712558-2243993		1.53	73 (*NOC2L, TNFRSF18, TNFRSF4*)
1p13.1	116168951-117581500	1.4		21 (*CD9P1, TRIM45*)
2pter-p25.3	6477-3368813		3.4	19 (*TSSC1*)
2q11.2-q13	100018907-112693871	12.7		129
2q14.1	114650166-115262744	0.6		1 (intron of *DPP10*)
2q14.1	115268926-115418974		0.15	1 (intron of *DPP10*)
2q14.1-q21.3	115425158-136187596	20.8		166
2q22.1-q23.3	137775162-153625228	15.9		61
2q23.3-q24.1	153631373-159375017	5.7		27
2q24.1-q24.3	159381356-165156442	5.8		42
2q24.3	165162652-165968804	0.78		6
2q24.3	167625090-169268884	1.6		9 (*STK39*)
3pter-p12.1	37570-85662543	85.6		718
4pter-p15.2	191-26362651		26.4	255 (*FGFR3*)
4p13	42031490-43193968		1.2	4
4p13-p11	43200181-49200003		6.0	37
4p11-qter	49275218-191262539	142		931
4q31.1	141506279-141625040		0.12	2 (*SCOC, CLGN*)
5pter-p15.31	68753-6212561		6.2	49 (*AHRR, TRIP13, TERT, NDUFS6, ADAMTS16*)
5q21.2	102968812-104225203	1.3		0
5q21.3	105381409-105843899	0.47		0
5q21.3-q22.1	107493790-110062750	2.6		11
5q23.1	118512522-121281380	2.8		12
5q23.2-q23.3	124356361-129125131	4.8		26
5q31.1-q31.2	133375174-136043822	2.7		36 (*TCF7*)
5q31.2	136231375-136300191	0.07		0
5q31.2-q32	136925251-144481375	7.6		158 (*WNT8A, EGR1*)
5q32-q33.1	145268956-148600233	3.3		31
5q33.2-q33.3	155168994-156318797	1.5		3 (*SGCD*)
5q34	166500163-166900243		0.4	1 (*ODZ2*)
5q35.2-qter	176325212-180650172		4.3	129 (*FGFR4, SCGB3A1, FLT4*)
6q22.33	129843975-130381431		0.54	4
8q24.12-qter	121625189-146262725	24.62		210 (*HAS2, MYC*)
9p24.1	8412749-8731371	0.318		2 (*PTPRD*)
9q34.11	129650050-130125185	0.475		20 (*NAIF1, CIZ1*)
9q34.3	140012561-140225027	0.213		3 (*CACNA1B*)
10pter-q22.1	87523-71568878	71.6		585
11pter-p15.4	187565-7193965	7.0		298
14q12-qter	25075026-106356252	81.3		1049 (*IGH*)
15q11.2-qter	20262522-100281493	80		1084
16pter-p13.3	53-5456277	5.5		267
17pter-p12	18890-15187519	15.2		334 (*TP53*)
19p13.3-p13.11	2250072-19137619	19.1		586
19p13.11	19143788-19306349		0.16	9
20q11.1-q13.32	28125216-56612507		28.5	408
20q13.32-qter	57343940-62387649	5.0		110
21q22.13-q22.3	38293990-45156291		6.9	120
22q12.3	31618959-34862722	3.3		23
22q13.1	36256330-37868873		1.6	55
22q13.1-qter	37975088-49581355	11.6		207

## Discussion

**Figure 4:**
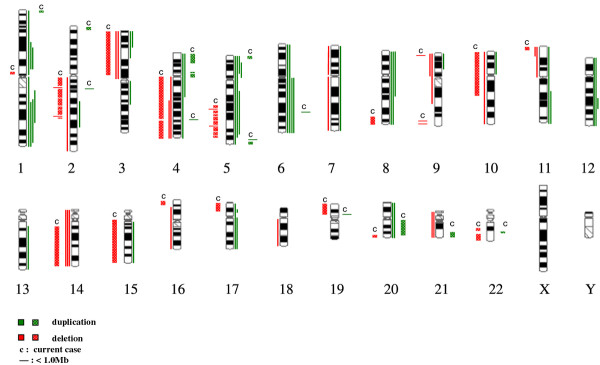
**Idiograms of the aCGH results from our case along with six cases using conventional CGH previously reported by Sowery et al.** Our current case is labeled as “c” and gains of our case are presented by green vertical patterned lines. Losses of our case are presented by red vertical patterned lines. “—” represent segments of losses or gains smaller than 1 Mb. For Sowery et al.’s study, gains are demonstrated by green vertical lines to the right of the chromosome idiograms; losses are demonstrated by red vertical lines to the left.

To compare our aCGH findings with previous conventional CGH data generated by Sowery et al. [8], we summarized our current case and their six cases of UESL into a chromosome idiogram (Figure 4). Our investigation of this single UESL case identified multiple unreported genomic imbalances involving gains of regions on chromosome 2 (2pter-p25.3 and 2q14.1), 19p13.11, 21q22.13-q22.3, and 22q13.1, as well as deletions of 1p13.1, alternating deletions on 5q (5q21.2-5q33.3), 8q24.12-qter, 9q (9q34.11 and 9q34.3), 15q11.2-qter, 16pter-p13.3, 17pter-p12, 19p13.3-p13.11, 20q13.32-qter, and 22q (22q12.3 and 22q13.1-qter). In addition to these regions mentioned above, twelve non-copy number variants, either segmental losses or gains less than 1 Mb were also identified on different chromosomes. Eight of these twelve smaller segments distributed on chromosome 2, 4, 5 and 19 were either adjacent to larger genomic imbalanced segments or embedded in the middle of the alternating multi-deletion regions. These smaller segmental changes are more likely due to the chromosomal instability. Four out of twelve smaller segments, located on chromosome 6 and 9, were isolated independently from other genomic imbalanced regions, and at least one of these regions on 9q contains tumor-related genes (*NAIF1* and *CIZ1*). We also identified imbalanced regions that overlapped with regions reported by Sowery et al., i.e., 3p (loss), 4p (gain), 11p (loss), 14q (loss), and 20q (gain).

**Figure 5:**
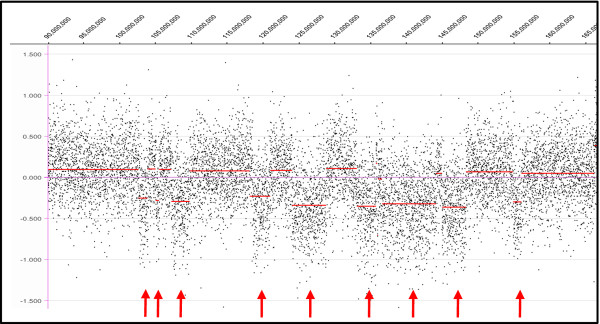
**Array-CGH result of alternating multiple segmental deletions on chromosome 5q of the current case using NimbleGen SegMNT.** Red arrows indicate the loss of chromosome material.

Our case showed more loss, approximately 645.3 Mb in total genomic size, compared to the total gain of 87.4 Mb. However, in Sowery et al.’s study, the opposite patterns of loss vs. gain were found, i.e., more gain than loss was shown using visual observation of the idiogram (summarized in Figure 4). A few imbalanced regions in our case are worth emphasizing. Alternating, multiple segmental deletions within the 5q21.2–5q33.3 region were detected (Figure 5). The sizes of the alternating deleted segments ranged from 69 kb to 7.6 Mb. It is impossible to detect these deletion patterns with routine cytogenetic techniques or conventional CGH. The results from the aCGH illustrated that the chromosomal instability in tumors is more complicated than a simple deletion, duplication or translocation. This phenomenon of alternating multiple segmental deletions has been reported in other types of solid tumors [13-15]. The same is true for the deletions on chromosome 8q (8q24.12-qter) and 17p (17pter-p12) detected in our study as opposed to the duplications reported by Sowery et al. We proceeded with a mutation assay of the *TP53* gene located on chromosome 17p. A missense mutation R248W of the remaining allele was found. To our knowledge, this is the fourth UESL case reported to carry the *TP53* gene mutation [10,11]. This particular mutation, as a somatic or germline change, has been reported in a variety of solid tumors [16]. Interestingly, all these mutations, identified in cases with UESL, namely, K120M [11], V216M [11], S245G [10], and R248W (our case) are located in the sequence-specific DNA binding domain (amino acid residues 102–292 out of the 393 of the p53 protein), where the hot spots of mutations are located [17]. The major consequence of these mutations in this domain is loss of sequence-specific DNA binding to the canonical p53-binding site and loss of the ability to induce cell cycle arrest or apoptosis. It has been reported that ellipticine may restore the DNA binding domain and transcription function in transfected p53 mutants cells with different mutations in this sequence-specific DNA binding domain [18]. This could lead to a potential targeted therapy of UESL *via* restoring the biological activity of p53.

Three out of six patients in Sowery et al.’s study showed a loss of the whole long arm of chromosome 14. In our case, a partial deletion of the long arm (14q12-qter) was detected. This could be a pure deletion of the long arm of chromosome 14 at the breakpoint 14q12, or it could be due to an unbalanced translocation with another partner chromosome. Although loss of chromosome 14 has been reported in rhabdomyosarcoma and neuroblastoma and associated with poor outcomes in clear-cell renal cell carcinoma [19-21], it is unclear whether the loss of chromosome 14 plays an important role in UESL initiation or progression. In Sowery et al.’s assay, three out of six cases presented a gain of 5p with an overlapped region of 5pter-p13.3. Our case also had a gain of the short arm of chromosome 5, but the size was reasonably smaller, of only 6.2 Mb. In the literature, a gain of 5p has been found in osteosarcoma and malignant fibrous histiocytoma [22], and studies combining aCGH and gene expression assays suggest that 5pter-p15.3 harbors several candidate oncogenes including *TRIP13, TERT, NDUFS6,* and *ADAMT16 *[13,14]. A gain of 4p with an overlapped region of 4pter-p15.2 was detected by our study and Sowery’s group. FISH using the LSI LGH/FGFR3 probe revealed an amplification of the *FGFR3* gene in this region (Figure 2C). *FGFR3* amplification and/or overexpression was reported in rhabdomyosarcoma and bladder cancer [23,24]. These recurrent findings may be critical to the development of the malignant phenotype or associated with sarcoma. Losses of segments on 3p and 11p were shared by both studies, but Sowery’s group also detected gains in these regions. This phenomenon could be due to the secondary chromosomal changes.

In the literature, multiple karyotypic analyses reported that UESL frequently harbors t(11;19)(q11;q13.3/13.4) [6,7,25]. However, this was not noted either in our case or in Sowery et al.’s study. UESL shares similar clinical and histological features and a genetic profile with mesenchymal hamartoma of the liver (MHL). Over the past few years, three cases of UESL have been reported to develop within MHL, displaying similar genetic rearrangements involving either an add(19)(q13.4) or t(11;19)(q11;q13.3-13.4), nearly identical to the genetic rearrangements observed in previous solitary cases of MHL [6,7,25]. Rajaram et al. have postulated that the t(11;19) translocation is likely related to the development of solitary cases of MHL, but for progression of MHL to UESL, additional genetic alterations at other loci are required [9].

Based on our high-resolution aCGH data, numerous genes were located in the imbalanced regions, and some of them were tumor-related genes. In addition to the genes mentioned above, several notable genes that are worth further investigation are listed in Table 1.

In conclusion, although numerous chromosomal abnormalities and tumor-related genes were identified in this case, the tumorigenic mechanism of UESL is still unclear. Further investigations are required; however, the clinical diagnosis of UESL is difficult because there are limitations associated with determining morphological classifications. The effort to find genetic markers to subclassify UESL is important, and genomic profiles will assist in current clinical practices. In addition, we also emphasized the inactivation of *TP53* gene through the loss of heterozygosity and a pathogenic mutation of the remaining allele. Restoration of *TP53* gene function could be of interest for therapeutic strategies of UESL. Our findings shed new light on the clinical diagnosis and add strong evidence of a potential targeted treatment.

## Consent

Written informed consent was obtained from the patient for publication of this case report and any accompanying images. A copy of the written consent is available for review by the Editor-in-chief of this journal.

## Abbreviations

UESL: Undifferentiated Embryonal Sarcoma of the Liver; FISH: Fluorescence In Situ Hybridization; aCGH: array-CGH; AST: Aspartate Aminotransferase; ALT: Alanine Aminotransferase; EMA: Epithelial Membrane Antigen; SMA: Smooth-muscle Actin; MRI: Magnetic Resonance Imaging; MHL: Mensenchymal Hamartoma of the Liver.

## Competing interests

The authors declare that they have no competing interests.

## Authors’ contributions

XH carried out the DNA sequencing analysis, interpreted the aCGH data and drafted the manuscript. HC and XW performed the aCGH analysis. MJ made the pathologic diagnosis and prepared the tumor sample. WX designed the primers for DNA sequencing analysis. RZ performed the FISH analysis. JL supervised the aCGH and FISH analysis. SL and JN conceived this study and helped draft the manuscript. All authors read and approved the final manuscript.
